# Participant concerns for the Learner in a Virtual Reality replication of the Milgram obedience study

**DOI:** 10.1371/journal.pone.0209704

**Published:** 2018-12-31

**Authors:** Mar Gonzalez-Franco, Mel Slater, Megan E. Birney, David Swapp, S. Alexander Haslam, Stephen D. Reicher

**Affiliations:** 1 Microsoft Research, Redmond, Washington, United States of America; 2 Department of Computer Science, University College London, London, England, United Kingdom; 3 Department of Clinical Psychology and Psychobiology, University of Barcelona, Barcelona Spain; 4 Applied Psychology Department, University of Chester at University Centre Shrewsbury, Shrewsbury, England, United Kingdom; 5 School of Psychology, University of Queensland, Brisbane, Australia; 6 School of Psychology and Neuroscience, University of St. Andrews, Fife, Scotland, United Kingdom; Middlesex University, UNITED KINGDOM

## Abstract

In Milgram’s seminal obedience studies, participants’ behaviour has traditionally been explained as a demonstration of people’s tendency to enter into an ‘agentic state’ when in the presence of an authority figure: they attend only to the demands of that authority and are insensitive to the plight of their victims. There have been many criticisms of this view, but most rely on either indirect or anecdotal evidence. In this study, participants (n = 40) are taken through a Virtual Reality simulation of the Milgram paradigm. Compared to control participants (n = 20) who are not taken through the simulation, those in the experimental conditions are found to attempt to help the Learner more by putting greater emphasis on the correct word over the incorrect words. We also manipulate the extent to which participants identify with the science of the study and show that high identifiers both give more help, are less stressed, and are more hesitant to press the shock button than low identifiers. We conclude that these findings constitute a refutation of the ‘agentic state’ approach to obedience. Instead, we discuss implications for the alternative approaches such as ‘engaged followership’ which suggests that obedience is a function of relative identification with the science and with the victim in the study. Finally, we discuss the value of Virtual Reality as a technique for investigating hard-to-study psychological phenomena.

## Introduction

While the findings of Milgram’s famous Yale Obedience studies [1, 2] remain compelling after half a century, his explanation of these findings has become increasingly unconvincing. Milgram deceived his participants into participating as a Teacher in a learning experiment. This involved giving a Learner (a male confederate of Milgram’s) a list of word pairs, then going through the list, each time giving the first word of the pair and asking which of four options it had originally been paired with. Participants (Teachers) were instructed to inflict an escalating series of electric shocks on the Learner each time he made an error (though in fact the shocks were not real).

In the best-known variant of the study (the so-called baseline condition) the Learner was not physically present but could be heard giving various pre-scripted indications of pain and dissent at different shock levels, played from a tape recorder. In this case, some two-thirds of participants (65%) went all the way to the maximum shock level—an apparently lethal 450v. These studies on obedience to authority ‘shocked the world’ [3].

In his early publications, Milgram advanced various potential explanations for his findings. However, in his summary text *Obedience to Authority*, written roughly a decade later in 1974, he settled on the so-called ‘agentic state’ account [4]. This drew heavily on his interpretation of the notion of ‘the banality of evil’ [5]. It proposed that people have an inherent tendency to become so focussed on doing what is demanded of them by an authority figure as effectively and efficiently as possible that they lose sight of the consequences of their actions. In short, toxic obedience is seen to stem from the fact that people are unaware of the harm they are doing.

There are many reasons to doubt this account [6], including its inability to explain patterns of obedience and disobedience. Indeed, Milgram conducted around 30 versions of his basic paradigm (though not all were published, see [3]) in which levels of obedience ranged from 0% to 100%. Such a large variation in findings cannot be accounted for by the agentic state explanation [7].

However, potentially the most compelling evidence against the ‘agentic state’ explanation—and the core assumption that participants are inattentive to the Learner—has more to do with what happens *while* people obey rather than with *whether* they obey. Milgram’s own film of his studies, *Obedience* (1965), suggests that participants are deeply concerned with the plight of their victim and deeply ambivalent about what they are doing. Milgram [4] himself notes how his procedures ‘created extreme levels of nervous tension in some Ss’, [2]: nervous laughter, sweating, trembling, stuttering, even groaning. He notes how participants would sometimes make the shocks as brief as possible and would also demonstrate discomfort by hesitating to press the shock button, especially later in the studies when the shock levels were higher and when the Learner protested more vociferously [3]. There also appear to have been instances where participants tried to cue the Learner in to the correct response through the use of emphasis when they read out the word choices in each trial [8].

The first aim of this paper is to provide systematic data to back up these anecdotal observations. Do participants demonstrate concern for the Learner even as they administer shocks in the Milgram paradigm? Do they try to help him answer the memory test correctly and so avoid being shocked? More concretely, do they place greater verbal emphasis on the one correct answer when reading it out than on the three incorrect answers? If this can be demonstrated, it would provide strong and direct evidence against the idea that people shock because they are unaware of and unconcerned with the plight of the Learner.

But if participants are concerned for the Learner why do they still obey experimental instructions? What leads them to privilege the demands of the Experimenter to continue administering shocks over the demands of the Learner to stop? One answer, which Milgram himself alludes to in his unpublished experimental notebooks, is that this is a function of identification. In general terms, the more one sees oneself having a positive relationship with the source of an authority, the more likely one is to obey that authority. Specifically, a broad range of evidence supports the contention that the more participants identify with science, the more they obey the Experimenter’s instructions [9–11]. From this perspective, those who obey do so not through unawareness but because, whatever other concerns they have, they ultimately consider it the right thing to do. That is, obedient participants are *engaged followers* [12, 13].

In light of this debate, a second aim of this paper is to explore the relationship between identification with science (IS) and concern for the Learner (CL) while shocks are being delivered. This is measured by (a) the emphasis laid on the correct response so as to help participants avoid incorrect answers, (b) the hesitation in applying shocks should an incorrect answer be given (especially at higher shock levels), and (c) reported levels of stress when administering shocks.

Here we do not have a single prediction, but rather explore two alternative possibilities. The most straightforward is that there will be a *negative* relationship between IS and CL. That is, the more a participant identifies with science and is concerned with the successful completion of the study, the less they care about the experience and fate of the Learner—more concretely, the less they would try to help him avoid shocks, the less they would hesitate in applying such shocks, and the less stressed they would be by doing so.

However, perhaps more intriguingly, it is also possible that there will be a *positive* relationship between IS and CL. That is, science demands concern for all those involved in the investigatory process, including research participants. Hence increased salience of scientific identity would not only increase commitment to the Experimenter and to the study but also increase concern for the Learner. This would increase the extent to which participants would help the Learner avoid shocks, increase the hesitation before applying shocks, and increase the stress of doing so (even if, ultimately, their commitment to completing the study would prevail).

### Methodological and ethical issues

Anyone seeking to revisit Milgram’s studies of obedience faces a dilemma. How can we do this when Milgram’s methods are ethically unacceptable? To resolve this issue, researchers have developed a range of solutions that include stopping the shocks before they get too extreme [14], using less dramatic analogues of Milgram’s paradigm [10, 15, 16] and using actors [17]—see [6] for a review.

In the present study, we address these ethical concerns through the use of Virtual Reality (VR). A previous VR simulation of the obedience to authority paradigm [18] has shown that participants experience the paradigm in ways that mirror the experience and behaviour of those in Milgram’s original studies—albeit with somewhat lesser level of intensity and higher absolute levels of obedience. We now use a revised and updated VR simulation in order to investigate the processes involved in Milgram’s paradigm—specifically the issues discussed above concerning participants’ stress levels, helping behaviour, and their identification with authority.

The study recruited students from a London University as participants. We used a real Experimenter and a virtual character as a Learner representing a student, wearing a shirt with the university’s insignia. In this way, identity as a student was kept constantly and chronically salient throughout the study. We used a priming procedure either to emphasize this salient identity or else to emphasize science in order to manipulate participants’ level of identification with Science. In effect then, we expected no difference in identification with students between conditions, but a difference in identification with Science. Accordingly, then, we label these conditions ‘Science’ and ‘Non-Science’.

Participants then went through a modified version of Milgram’s classic ‘proximity’ study variant (see [4]) in which the Learner and the Teacher are in the same room. In addition, we ran a control condition in which people went through the procedure of reading out the words for each trial within the VR environment but without a virtual Learner and hence without imposing shocks.

Unlike most Milgram studies, where the focus is on levels of obedience (which we ourselves did measure through number of shocks administered), our primary interest lies in levels of concern for the Learner while people obey (and, in this regard, the higher levels of obedience in VR paradigms are an advantage for our purposes). We measured concern through (a) helping behaviour (i.e. the extent to which the correct answer on each trial was pronounced louder than the other three—incorrect—answers); (b) the delay between receiving an incorrect answer and administering a shock, and; (c) self-assessed somatic arousal (indicating stress).

Our key hypotheses were as follows. First, we expected that participants as a whole would attempt to help the Learner by pronouncing the correct answer to each trial more loudly than the incorrect answers in the experimental conditions relative to the control condition (H1).

Second, we investigated two alternative hypotheses concerning the relationship between identification with science and the three measures of concern for the Learner (helping, delay, and stress). According to H2a identification with science should decrease concern. According to H2b it should increase concern.

The experiment received approval from the Research Ethics Committee at the University where the experiment took place. Participants took part in the study with full written consent. Participants were told that they could withdraw at any moment, without giving reasons, and without losing any benefits to which they would otherwise be entitled. They were informed about this several times, in writing and verbally. Following Milgram’s protocol, if participants hesitated to respond, they were told to “Please continue” by the Experimenter. However, for ethical reasons, participants who continued to resist were not given the other three prods. Six participants (out of 40) asked to quit, and, when they did, the experimental session was terminated.

## Method

### Participants and design

Forty students enrolled at a University in London took part in the experimental study. Participants were male, (*M*^*age*^ = 20.85; *SD* = 1.77) and were recruited via an email advertisement sent to students across the university. In order to reduce the chance that participants would be familiar with Milgram’s obedience studies, postgraduates and students studying psychology were not sent this advert. The study had a between-subjects design with two experimental conditions (identification with science—Science—vs. no identification with science—Non-Science). In the second part of the study, which consisted of a control group, an additional 20 males (*M*^*age*^ = *26*.*30*, *SD* = 2.45) from the same university took part.

### Apparatus

A four-screen projection ‘Cave’ system [19] was used for generating the Immersive Virtual Environment. Participants were fitted with Crystal Eyes shutter glasses that were synchronized with the projectors, delivering active stereo at 50 Hz each eye. Head-tracking was performed with an InterSense IS-900 tracking device so that the rendering display was updated per the participants’ head orientation and position. The program was written using the Unity3D programming platform [20]. The audio was recorded from the Audacity software using a TOA WM-370 microphone connected to a wireless condenser (Beyerdynamic Diversity Receiver S250).

### Procedure

A visual of the setup can be found in S1 Fig. As participants arrived at the laboratory they were randomly assigned to either the Science or Non-Science conditions (see S1 Text). In all cases, participants were given an information sheet that explained that the experiment consisted of a training scenario, in which a virtual human character was required to learn a series of word-pairs. Participants were then required to test whether the virtual Learner had remembered these word-pairs. When he had not (i.e. he made an error) they were told that they should administer virtual electric shocks to him. After hearing a description of the study, participants gave written consent, and were asked to complete a questionnaire consisting of demographic questions and items measuring baseline physiological arousal (i.e., stress levels). Then, depending on the experimental condition to which they had been allocated, participants were told that researchers were interested in their ideas about science (Science condition) or about being a student in London (Non-Science condition). This manipulation was modelled after a previously validated manipulation of social identification [21]. Participants were asked to list three things that are good about science (being a student), three things they have in common with scientists (students), and three policy changes that they would like to see to help scientists (students).

After completing the questionnaires, participants were seated on a chair by a desk inside the Virtual Reality Cave room. A real shock machine apparatus was placed on the desk in front of them (S1 Fig and S1 Video). Facing them was a male virtual human character (i.e., the ‘Learner’). In all cases the virtual character wore a sweatshirt bearing the university’s insignia.

Participants were then told that they should read out a sequence of five words that would appear on the wall behind the Learner. The first word represented the cue and the Learner was required to answer with one of the four subsequent words, which represented the word associated with the cue word. This was the Learner’s memory task. Only one of the four words was correct, and this was indicated to the participant in capital letters. For example, the five words might be: *book*—*read write title MARK*, where ‘*book*’ is the cue word, and the correct answer is ‘*mark*’.

If the Learner responded with the correct word, participants were instructed to say out loud “Correct”, and then automatically the next word sequence would appear. If the Learner made the wrong choice, participants were instructed to reply “Incorrect. The correct answer is___” and then administer a ‘shock’ to the Learner by pressing a button using the (fake) shock machine on the desk in front of them. Each time a mistake was made, the participant was asked to increase the ‘voltage’ of the shock by turning a button on the shock machine. Automatically the next word sequence would appear and the process would begin again. The study consisted of 30 trials, and on 10 of these the Learner gave the correct answer. Consequently, there were 20 levels of shock in total.

As much as possible, the script used by the ‘Learner’ and the Experimenter was based on the script used by Milgram in his baseline study (see S1 Text). For instance, if a participant hesitated at any point during the experiment, the Experimenter would use Milgram’s first prod, ‘Please continue.’ If the participant continued to show reluctance, the Experimenter would ask the participant whether he would like to quit the study. If the answer was yes, the study ended immediately. If the answer was no, then the participant was again told to ‘Please continue.’

In addition, from Trial 11 the Learner’s responses rose in intensity of objection as the trials progressed (e.g., Trial 12: ‘I don’t want to continue!’). For instance, on Trial 16 the Learner said in response to the new set of words “Forget it, I’m not answering!”, “You have no right to keep me here, let me out!” At this point the Experimenter intervened by saying to the participant “Remember that if he doesn’t answer, the answer should be taken as incorrect”, to which the Learner responded “I don’t want to continue, don’t listen to her!” After that, the complaints and painful responses by the Learner to the shocks continued to increase. On Trial 28 the Learner fainted and stopped answering entirely. Nevertheless, participants continued to be provided with new sets of questions and asked to administer shocks through to Trial 30. These scripted responses were intended to simulate the authority aspect of the original Milgram setup, while guaranteeing the right of the participants to exit the study. See S1 Video for a detailed example of the experimental procedures.

Upon completing the final trial (or quitting the study), participants were asked to complete a questionnaire assessing post-experimental physiological arousal (i.e., stress levels) before being debriefed by the Experimenter. This involved a semi-structured interview in which they were asked about their perception of the study and told about the purpose of the research.

After the experimental study was completed, a control condition was run with another set of 20 participants, in order to assess whether any emphasis participants placed on the ‘correct’ word in the experimental conditions was due to helping behaviour or to the fact that this word was written in capital letters. Participants allocated to this control condition entered the same VR environment as those allocated to the experimental conditions, except there was no virtual Learner present and therefore no shocks were administered. Participants were asked to read the same sequences of words in the same order and format as participants in the main experiment, displayed in the same place in the same virtual room. As in the main experiment, their speech was recorded and sound level was calculated (see below).

### Response variables

#### Manipulation checks

In order to check whether our manipulation of identification with science was successful, and also whether identification as a student was constant across conditions, participants completed a questionnaire with 7 items measuring the former (e.g. ‘I identify with the goals of psychological science’), and a further 5 questions that assessed the latter (e.g. ‘I identify with other students living in London’). Polychoric principal components analysis was used to obtain a single overall identification score for each scale, which are *ysci* for Science and *ynsc* for Non-Science. Full scales and details of the analysis can be found in the S4 Text.

#### Stress (Autonomic perceptions questionnaire: APQ)

To gauge levels of stress, we administered a 24-item measure of self-perceived arousal questionnaire [22] both before (*APQpre*) and immediately after the experiment (*APQpost*). Higher scores indicate enhanced awareness of bodily sensations. Stress was assessed as the difference between these scores (*dAPQ* = *APQpost*–*APQpre)*, which has been shown to be positively correlated with anxiety, heart rate and skin conductance [23]. Questions assessed whether people experienced stress indicators, such as trembling or shaking, lack of concentration, and dizziness (see S2 Text).

#### Helping behaviour (Relative sound pressure level: RSPL)

The relative sound pressure level (*RSPL*) is a measure of the amount of stress placed on the correct word compared to the remaining words. Participants were recorded while reading out the cue words, and this audio was analysed with a Matlab program. First, the SPL of each individual word was extracted. The SPL is defined by Eq (1),

$$SPL=20*log(\frac{RMS(x)}{ref})$$ (1)

*RMS*(*x*) is the Root Mean Squared of the signal and *ref* is defined by the sound pressure in air, considered the threshold of human hearing, with the final value being in decibels (dB). The more negative the dB values the lower the voice. Next, the SPL of the correct word in the list of 5 was normalized by the mean SPL of the other 4 words. This yielded the *RSPL*.

#### Discomfort (time to shock)

The time between the point at which the virtual Learner completed his response and the point at which the participant administered an electric shock was automatically recorded on all trials as a behavioural measure of discomfort (labelled *time2Shock*; see [18]). Insofar as this is expected to be particularly acute as the Learner appears to be more affected by the shocks, this was important specifically for the final two shocks given by each participant (time to last two shocks: *Time2ShockLast2*).

#### Obedience

Although not a response variable of interest in the current research, we recorded how many trials (out of a maximum of 20) that participants completed.

#### Presence

Participants completed a measure to address the extent to which people experience a Virtual Reality simulation as real [24]. The purpose of administering this questionnaire was to check that level of presence was not different between the experimental groups (see S5 Text for more details).

### Analysis

A Bayesian analysis was carried out (see S1 Dataset and S1 Statsmodel). This was motivated by the fact that there are several response variables and using frequentist statistical inference would provide no satisfactory level of control of the overall significance level for multiple tests apart from heuristics. Instead, with the Bayesian approach we can treat all statistical relationships in *one set of simultaneous stochastic equations* and make probability statements about any group of parameters of interest derived from the joint probability distribution of all parameters. Table 1 shows the overall model.

**Table 1:** Likelihoods for the response variables.

Eq.	Likelihood	Statistical Model
1	${RSPL}_{i}\sim N(\mu_{SPL,i},\sigma_{SPL}^{2}$)	*μ*_*SPL*,*i*_ = *β*_*SPL*,0_ + *β*_*SPL*,1_ *X*_*i*_ *i* = 1,2, …, *n*
2	${dAPQ}_{i}\sim N(\mu_{APQ,i},\sigma_{APQ}^{2})$	*μ*_*APQ*,*i*_ = *β*_*APQ*,0_ + *β*_*APQ*,1_ *X*_*i*_ + *β*_*APQ*,2_ *RSPL*_*i*_ + *β*_*APQ*,3_ (*X*_*i*_.*RSPL*_*i*_) *i* = 1,2, …, *n*
3	$Time2ShockLast2\sim N(\mu_{Time,i},\sigma_{Time}^{2})$	*μ*_*Time*,*i*_ = *β*_*Time*,0_ + *β*_*Time*,1_ *time*2*shock*_*i*_ + *β*_*Time*,2_*X*_*i*_ + *β*_*Time*,3_(*X*_*i*_.*time*2*shock*_*i*_) *i* = 1,2, …, *n*
4	${ysc}_{i}\sim N(\mu_{ysc,i},\sigma_{ysc}^{2})$	*μ*_*ysc*,*i*_ = *β*_0_ + *β*_*ysc*,1_ *X*_*i*_ *i* = 1,2, …, *n*
5	${ynsc}_{i}\sim N(\mu_{ynsc,i},\sigma_{ynsc}^{2})$	*μ*_*ynsc*,*i*_ = *β*_*ynsc*,0_ + *β*_*ynsc*,1_ *X*_*i*_ *i* = 1,2, …, *n*
6	${RSPL\prime }_{i}\sim N(\mu_{SPL,i}^{\prime },{\sigma \prime }_{SPL}^{2}$)	$\mu_{SPL,i}^{\prime }=\mu_{SPL}+{\alpha_{SPL,1}Ns}_{i}+{\alpha_{SPL,2}Sc}_{i}$ *i* = 1,2, …, *N*

n = 40 (the main experiment only), N = 60 (including the 20 controls).

*X*_*i*_ = 0 (Non-Science), 1 (Science) (factor with two levels, *i* = 1,…,n)

*Ns*_*i*_ = 1 (Non-Science) 0 (otherwise); *Sc*_*i*_ = 1 Science) 0 (otherwise)

(factor with 3 levels: Non-Science, Science, Control (aliased to 0), *i* = 1,…,N)

#### Likelihoods

We use the notation ***y* ~*N***(**μ, σ**^**2**^) to denote that ***y*** has a normal distribution with mean ***μ*** and variance ***σ***^**2**^. The likelihoods and statistical models for the response variables are shown in Table 1.

- Eq. 1 *RSPL*: This simple linear model relates RSPL to the variation in the two conditions (Non-Science vs Science).
- Eq. 2 *dAPQ* = *APQpost*–*APQpre*. This relates dAPQ to the conditions with *RSPL* as the covariate.
- Eq. 3 *Time2ShocksLast2* (time last 2 shocks): The average time to administer the final two shocks by condition and the average time to administer all other shocks (*time2shocks*).
- Eq. 4 *ysc*: The extent to which participants identified with science as a function of condition.
- Eq. 5 *ynsc*: The extent to which participants identified with being a student as a function of condition.
- Eq. 6 *RSPL'*: This relates RSPL to the variation across the three conditions (Control vs Non-Science vs Science). Note that *RSPL'* is the same as *RSPL* for the values corresponding to Non-Science and Science.

#### Prior distributions

Prior distributions for all parameters on the right-hand side of the equations are Normal distributions (mean 0, standard deviation 10). Hence the 95% prior credible intervals for these parameters are between ±20. All the standard deviations σ have half Cauchy distribution on the positive line with parameters median 0 and scale 5, so that the prior 95% credible intervals are approximately between 20 and 125. The model was fitted using Stan library in Matlab (http://mc-stan.org/users/interfaces/matlab-stan).

#### Convergence

Four chains each with the number of simulations as 4000 were used and convergence was obtained, with all Rhat values equal to 1.

### Results

There were no systematic differences between the Science and Non-Science groups with respect to age, their experience with computer games, their knowledge of computer programming, their prior experience of Virtual Reality (See S6 Text; S2 Fig), or their prior knowledge of the Milgram Obedience paradigm (S3 Text; S3 Fig).

The overall results are in Table 2 which shows summary statistics from the posterior distributions of the parameters of Table 1. Note that these are derived from the overall joint distribution of all the parameters. In what follows, P(A) means the posterior probability of A.

**Table 2:** Summaries of the posterior distributions of the model.

Coefficient	Term	Mean	S.E.	2.5%	50%	97.5%	P(>0)
**RSPL**							
*β*_*SPL*,0_	Intercept	5.72	0.013	3.44	5.72	7.95	1.000
*β*_*SPL*,1_	Science	1.43	0.018	-1.73	1.44	4.63	0.810
*σ*_*SPL*_		5.21	0.007	4.19	5.15	6.60	1
**dAPQ**							
*β*_*APQ*,0_	Intercept	17.10	0.076	3.64	17.12	30.31	0.991
*β*_*APQ*,1_	Science	5.40	0.100	-11.89	5.44	22.84	0.728
*β*_*APQ*,2_	wordspl	0.94	0.011	-1.01	0.93	2.96	0.828
*β*_*APQ*,3_	Science×wordspl	-2.26	0.018	-5.38	-2.26	0.84	0.076
*σ*_*APQ*_		32.46	0.044	25.85	32.05	41.22	1
**Time2ShockLast2**							
*β*_*Time*,0_	Intercept	-4.49	0.022	-8.28	-4.50	-0.43	0.016
*β*_*Time*,1_	time2shock	2.26	0.003	1.71	2.27	2.78	1.000
*β*_*Time*,2_	Science	1.67	0.011	-0.33	1.67	3.65	0.951
*σ*_*Time*_		3.22	0.004	2.57	3.18	4.07	1
**ysc**							
*β*_*ysc*,0_	Intercept	-1.70	0.004	-2.46	-1.70	-0.95	0.000
*β*_*ysc*,1_	Science	1.33	0.006	0.25	1.33	2.38	0.992
*σ*_*ysc*_		1.67	0.002	1.34	1.65	2.10	1
**ynsc**							
*β*_*ynsc*,0_	Intercept	-0.98	0.004	-1.64	-0.98	-0.34	0.002
*β*_*ynsc*,1_	Science	0.01	0.005	-0.92	0.01	0.93	0.507
*σ*_*ynsc*_		1.48	0.002	1.18	1.46	1.87	1
**RSLP**^***'***^							
*μ*_*SPL*_	Intercept	3.88	0.011	1.88	3.88	5.90	1.000
*α*_*SPL*,1_	Non-Science	1.87	0.016	-1.00	1.85	4.74	0.904
*α*_*SPL*,2_	Science	3.27	0.016	0.43	3.26	6.16	0.988
$\sigma_{SPL}^{\prime }$		4.66	0.005	3.89	4.62	5.65	1

The factor Priming = 0 for Non-Science, and 1 for Science (i.e., *X* in Table 2). The intercept term corresponds to Non-Science when all covariates are 0. For each parameter the mean, standard error, and 2.5, 50, and 97.5 percentiles are given, and the posterior probability of the parameter being positive P(>0).

#### Manipulation check

S4 and S5 Figs show the effect of the manipulation on participants’ identification with either students or scientists. Although participants in the Science condition appeared to have higher identification with science, there was no such effect in the Non-Science condition on identification with students. Importantly, participants gave nearly identical responses on the ‘identification with students’ questionnaire, regardless of the manipulation. However, participants in the Science condition had greater median answers on all but one question. This can be seen also in S5 Fig, which shows nearly identical mean PCA scores *ynsc* (non-identification with science) and *ysci* (identification with science) on the identification with student score. However, on the identification with science items, the *ysci* score is greater than the *ynsc* score. Hence, there is a very high probability that the *ysci* scores are greater for the Science group than the Non-Science group (*P*(*β*_*ysc*,1_ > 0) = 0.992; see S4 Text). However, for *ynsc*, the equivalent probability is 0.5.

### Main analyses

#### Helping behaviour (re H1)

In support of H1, the evidence suggests that participants in the main experiment tried to help the virtual Learner. This can be seen by examining RSLP^*'*^, the sound pressure level between the participants and the controls (S6 Fig). The grand mean of the model μ_SPL_ is positive with probability 1.0. The probabilities for the coefficients of the Non-Science and Science factor being positive are 0.90 and 0.99 respectively.

#### Identification with science and concern for the Learner (re H2)

First, in relation to helping behaviour, from the joint parameter distribution, the probability is that the Science coefficient is greater than the Non-Science coefficient, *P*(*α*_*SPL*,2_ > *α*_*SPL*,1_) = 0.83. Also, S6 Fig suggests that the RSPL was higher for participants in the Science condition than the Non-Science condition, *P*(*β*_*SPL*,1_ > 0) = 0.81 (in the main experiment, excluding the Controls). This supports our H2b. That is, higher identification with science is associated with greater concern for the Learner.

Second, in relation to hesitation when administering shocks, S7 Fig shows the means of the difference between the time taken to administer the last two shocks minus the average time to administer all shocks. These data shows that overall participants in both the Science and the Non-Science group delayed administering the shocks in the latter part of the paradigm. However, those in the Science condition waited longer than those in the Non-Science condition. In Table 2 the equation for *Time2shocklast2* shows the dependency on the manipulation and the average time to shock (*time2shock*). The table shows that the probability of the coefficient of *time2shock* being positive is 1, but more interesting is the probability of the coefficient being greater than 1, P(*β*_*Time*,1_ > 1) = 1.0, P(*β*_*Time*,1_ > 1.815) = 0.95. Hence, other things being equal, the time to shock in the last two trials was greater than the time to shock overall. Moreover, as seen in Table 2, the probability is 0.951 that the waiting time is greater for participants in the Science condition compared to the Non-Science condition. This again supports H2b.

Third, in relation to reported stress, there is evidence that those in the Non-Science group had a greater *dAPQ* than those in the Science group—the respective means ± SE being 28.7 ± 8.62 and 15.8 ± 5.23 (Cohen’s d = 0.40, just less than medium). Table 2 shows that the intercept term is clearly positive (this corresponds to the Non-Science condition for any fixed level of RSPL). In contrast with the other measures, this is supportive of our H2a: concern for the Learner is greater in the Non-Science condition.

### Supplementary analyses

#### Identification with science and the relationship between helping and stress

Our main analyses suggest discrepant effects of identification with science on the various measures of concern for the Learner. Whereas identification with science increases helping and hesitation to deliver shocks, it decreases reported stress. One way to consider this is that the three measures of concern are not independent but interact and moreover, that this interaction is moderated by identification with science. That is, where there is high concern for the Learner, this generates higher levels of helping which in turn reduces stress. Conversely, where concern is lower there is no relationship between helping and stress. To be more concrete we would then expect a negative relationship between helping and stress (higher helping associated with lower stress) in the Science condition but not the Non-Science condition.

This is supported in our data. As shown in S8 Fig, the results suggest that there is no relationship between dAPQ and SPL for those in the Non-Science condition. However, in the Science condition the greater the SPL, the lower the post-experience stress as measured by APQpost. Indeed, the dAPQ entry in Table 2 shows that there is an interaction between RSPL and the priming. Compared to the Non-Science condition the slope of line fitting dAPQ on SPL is negative, with probability *P*(*β*_*APQ*,3_ < 0) = 1 − 0.076 = 0.924. Although we cannot infer the direction of causality, this finding is compatible with the idea that amongst the Science group, the more that they helped (by emphasizing the correct word) the less stress they experienced.

#### Obedience

In an earlier VR study [18] 6 out of 23 participants in a similar setup (though without any priming and without any attempt to persuade participants to continue) withdrew before giving all 20 shocks. In the present study 6 of the 40 participants withdrew early. Four of the 6 were in the Non-Science group.

## Discussion

This study addressed two questions. The first was whether participants, in a Virtual Reality reprise of one instance of the Milgram Obedience paradigm, display concern for the (male) Learner as reflected in the help they provide him by emphasizing the correct answers. The second was how this concern for the Learner—as reflected not only in helping but also in the hesitation to apply shocks and the level of stress provoked by doing so—is affected by identification with science. Would participants show more or less concern when such identification was accentuated?

The results relating to the first question are clear. Compared to a control condition in which no Learner was present, and participants simply read out the right answer on each of the 30 trials, those who were required to administer shocks to a virtual Learner (as per the Milgram paradigm) were more likely to emphasize the correct answer by reading it out more loudly than the incorrect answers (supporting H1). That is, even as they continue to impose electric shocks, participants do attend to the Learner and seek to help him avoid being shocked. It seems, then, that people do not apply electric shocks *because* they cease caring about the Learner, but rather *despite* the fact that they care.

The results relating to the second question are, at first glance, more mixed. On the one hand, the levels of help and the hesitation in applying shocks is greater where identification with science is made salient, supporting our H2b. Conversely, the level of stress is greater when identification with science is not made salient, supporting our H2a. As we suggest, however, one way of making sense of this apparent contradiction is to examine the interactions between our various ‘concern’ measures and the way that this depends upon levels of identification with science.

Thus, it is plausible to expect that if people do something to help the Learner, such as cueing them in to the right answer, and if the Learner still gets it wrong, then they (not you) become accountable for their plight. This is consonant with what we know about victim blaming in general [25, 26]. It is also consonant with findings from other research into the Milgram paradigm which suggest (a) that participants become increasingly keen for Learners to get the answer correct as shock levels increase and (b) that if the Learner continues to make errors, participants can become exasperated with them [8]. What is more, one might also plausibly expect such an impact of helping on stress precisely where one feels more concern for the Learner and hence questions of accountability for his plight are more acute. Assuming, then, that such concern is higher when science identity is salient, this would account for our finding of a (negative) relationship between helping and stress in the Science condition and of no such relationship in the Non-Science condition. It would explain why, in the Science condition, participants help more, hesitate more and, as a consequence, feel less rather than more stressed.

In sum, considering the complexity of relations between our concern measures, all the findings can be interpreted as consonant with our H2b: that identification with Science increases concern for the Learner. As we have argued, this can be explained by the fact that the Learner and the participant are part of the same scientific study and, especially when science identity is made salient, are more likely to be seen as members of a common ingroup and more likely to benefit from norms of concern for all those who take part in an experiment.

But what might be more surprising is that those high science identifiers who, across a range of studies, have been shown to be most likely to obey the Experimenter in shocking the Learner [9–11, 13] are also those who are most concerned for the Learner’s plight. This implies that obeying the Experimenter and caring for the Learner are not necessarily counterposed. Not only can one be—and participants are—concerned for both, but an increased tie to the Experimenter can be associated with an increased concern for the Learner.

Of course, such an interpretation of our findings has to be tentative, given that it is based on a post-hoc interpretation of our findings. What is not in doubt, however, is that obeying the Experimenter and showing concern for the Learner clearly co-exist and also that identification with science is an important moderator of both helping and stress in a Milgram-like paradigm. We are therefore in a position to draw two important lessons from our findings. One concerns Milgram’s agentic state account, premised on the lack of attention of the participant to the plight of the Learner. Even within a VR simulation (where participants know that no real harm is actually being done), participants are aware of the Learner’s suffering, reported being stressed by it, and help to try to prevent it. Previous evidence relating to this point was either indirect (showing that ‘inattentiveness’ as an explanation had problems in explaining various facets of Milgram’s findings) or else anecdotal (for a review see 11). We now have experimental evidence that Milgram’s ‘agentic state’ explanation of his own findings is incorrect.

The other lesson concerns our own alternative ‘engaged followership’ account of Milgram’s findings. As would be expected from this perspective, the extent to which one identifies with the science of the study plays an important part in determining levels of stress and helping. To date, this account has been mainly used to explain commitment to the study and hence levels of obedience to experimental instructions. Here we add an extra dimension by addressing the participant’s relationship to the Learner as well as the Experimenter. We certainly do not claim that our findings constitute support for ‘engaged followership’—not least because we did not have specific a priori hypotheses as to how identification with science would impact concern for the Learner. Our aim was more exploratory—to see whether identification would have effects and, if so, what they are. While the patterns proved to be more complex than we had anticipated, we certainly consider that we now have grounds for exploring further the way in which identification with science affects the various relationships in Milgram’s paradigm—those between participant and Learner as well as between participant and Experimenter (as suggested by [11, 12]).

There is also a third lesson which we wish to draw from this study. One of the great methodological challenges for psychology is to find ways of studying dramatic and impactful phenomena (such as toxic obedience) in ways that are both rigorous and ethical. Virtual Reality has been proposed as one potential solution [27–30] and indeed previous research shows that people experience and respond to a VR simulation of the Milgram paradigm in ways that are akin to the original [18]. This research goes further in showing that VR methodology is particularly valuable as a means of investigating relations between variables and hence elucidating psychological processes. Indeed, it is the first study in the context of the Milgram obedience paradigm in which VR has been used in this way, exploring the respective merit of ‘agentic state’ and ‘engaged followership’ models of obedience to authority. As in any research, there is a need to triangulate the present findings with others using different methods. Nonetheless, we would suggest that the strength of these findings points to the value of VR in providing rigorous data concerning hard-to-study phenomena. Moreover, the use of VR has made it possible to study the impact of individual differences amongst participants in order to understand the psychological components of the tendency to follow the Experimenter’s instructions (see [31] for a discussion).

To conclude, then, this study challenges the idea that people follow the instructions from an authority figure because they are unaware of or uninterested in the impact upon their victims. It shows that they are attuned to both the authority and the victim and that they seek to do their best by both. Participants strive to reconcile the contradictory demands placed upon them by the Experimenter and the Learner, inflicting shocks as demanded by the former while trying to help the latter avoid such shocks. It may be of little consolation to those on the receiving end, but our findings suggest that, in some circumstances at least, perpetrators can care for their victims even as they harm them.

## Supporting information

S1 Fig Visual of the Virtual Reality paradigm. A participant in the Cave faces the Learner. In Figure A the ‘Learner’ is the virtual male character wearing a ‘UCL’ sweatshirt. Behind the character are the cue word and 4 response words. The participant is seated at a desk, and his right hand is turning up the voltage on the shock machine. Figure B shows the scene photographed from outside the Cave. (The words and images are blurred because the Cave displays a pair of stereo images that are separated by the stereo glasses).
(TIFF)

S2 Fig Familiarity with computers. Box plots of responses to how familiar participants were with computer use, programming, and prior experience of VR, where 1 = Not at all and 7 = very much so.
(TIFF)

S3 Fig Familiarity with Milgram. Box plot showing participants’ level of familiarity with Milgram’s obedience studies across conditions. The effect size comparing Science with Non-Science is .047, indicating no difference between groups.
(TIFF)

S4 Fig Responses to the identification manipulation. Box plots of responses to the manipulation of (a) the identification with students (i.e. non-science) questionnaire and (b) the identification with science questionnaire. The medians are the thick horizontal lines and the boxes show the interquartile ranges (IQR). The whiskers extend from max (min value, lower quartile—1.5*IQR) to min (max value, lower quartile + 1.5*IQR). Points outside of this are shown individually.
(TIFF)

S5 Fig Descriptive statistics of responses to the identification manipulation. Bar charts showing the means and standard errors of the combined priming questionnaire score derived from the Polychoric PCA over the questionnaire scores.
(TIFF)

S6 Fig Descriptive statistics of RSPL scores. Bar chart showing mean ± SE of RSPL; (A) By the Control and Experimental (Science, Non-Science) groups (B) Distinguishing the Science and Non-Science groups.
(TIFF)

S7 Fig Time taken to shock. Difference between experimental conditions on the measure ‘timetoshock’.
(TIFF)

S8 Fig Relationship between helping and stress. Scatterplot illustrating the relationship between helping and stress by experimental group.
(TIFF)

S1 Text Avatar script. Procedure used for randomising participants to conditions and the script followed by the avatar (i.e. the ‘Learner’).
(PDF)

S2 Text Stress measure. Items and procedural information relating to the APQ stress measure.
(PDF)

S3 Text Familiarity with Milgram. Items measuring participants’ levels of familiarity with Milgram’s obedience studies.
(PDF)

S4 Text Manipulation check. Explanations, items, and analysis of the questionnaire used to assess the efficacy of the priming. (Table 1) Items used to assess priming efficacy. (Table 2) Correlations for priming scores.
(PDF)

S5 Text Presence. Background information, items, and effect sizes of presences measures. (Table 1) The full set of presence questions and effect sizes comparing the two groups.
(PDF)

S6 Text Familiarity with technology. Items and effect sizes for items measuring participants experiences with gaming, computers, programming, and VR. (Table 1) Questions and their effect sizes.
(PDF)

S1 Video A video showing the experimental paradigm. (MP4)

S1 Dataset Data collected from participants. (XLS)

S1 Statsmodel Program used to run the analysis. (PDF)
