# Supplementary figures and images for: Participant concerns for the Learner in a Virtual Reality replication of the Milgram obedience study
Source: PLoS One. 2018 Dec 31;13(12):e0209704. doi: 10.1371/journal.pone.0209704 (PMC6312327; doi:10.1371/journal.pone.0209704)

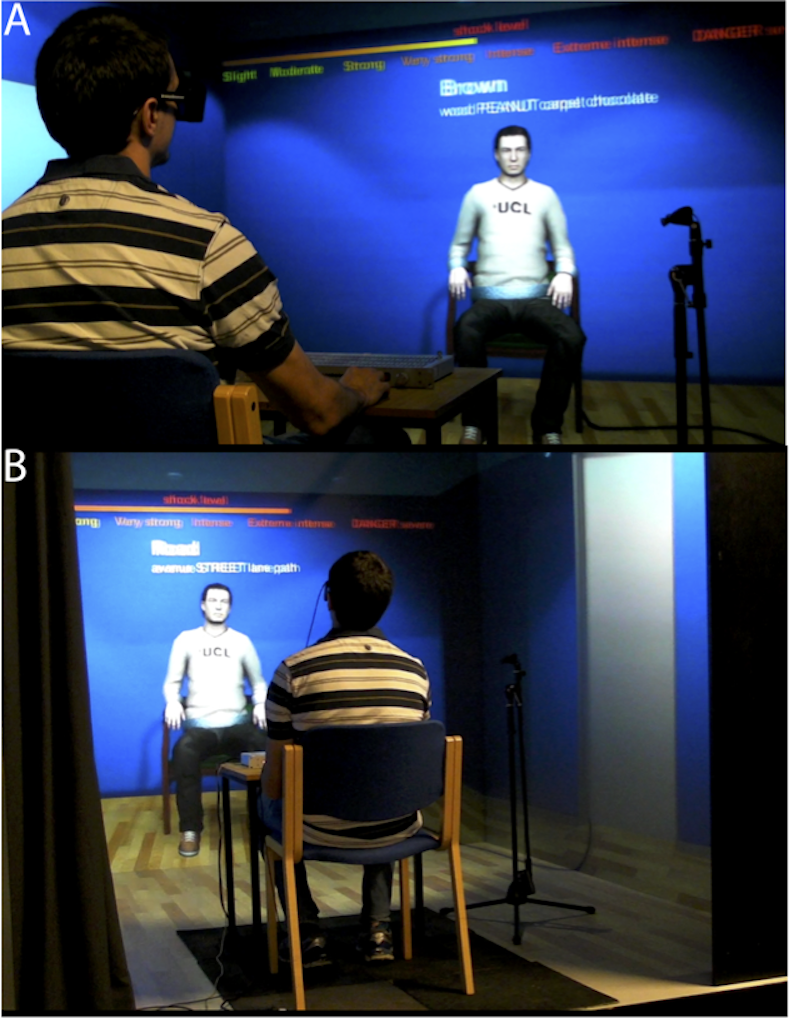

Supplement: S1 Fig — A participant in the Cave faces the Learner. In Figure A the ‘Learner’ is the virtual male character wearing a ‘UCL’ sweatshirt. Behind the character are the cue word and 4 response words. The participant is seated at a desk, and his right hand is turning up the voltage on the shock machine. Figure B shows the scene photographed from outside the Cave. (The words and images are blurred because the Cave displays a pair of stereo images that are separated by the stereo glasses). (TIFF) [file pone.0209704.s001.tiff]

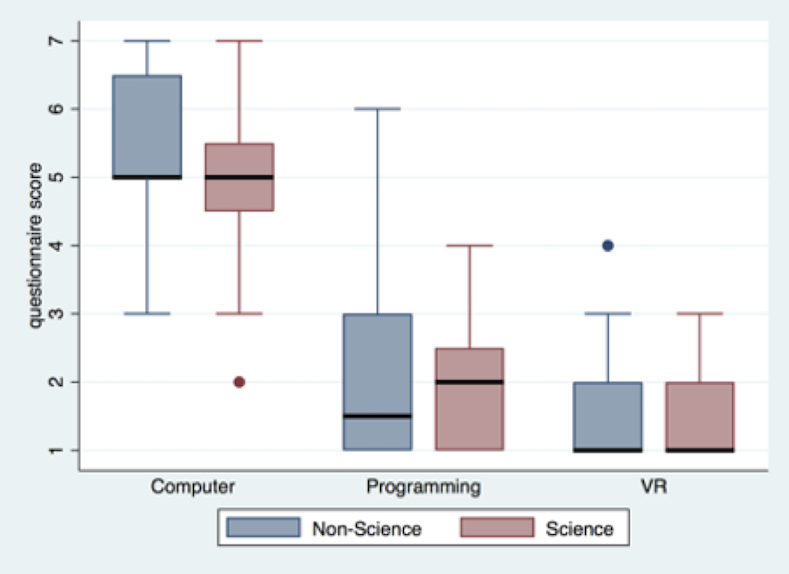

Supplement: S2 Fig — Box plots of responses to how familiar participants were with computer use, programming, and prior experience of VR, where 1 = Not at all and 7 = very much so. (TIFF) [file pone.0209704.s002.tiff]

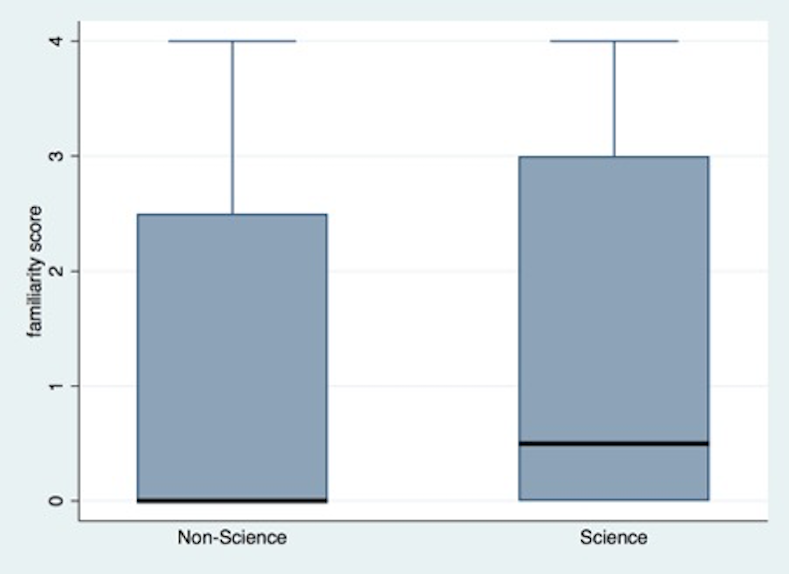

Supplement: S3 Fig — Box plot showing participants’ level of familiarity with Milgram’s obedience studies across conditions. The effect size comparing Science with Non-Science is .047, indicating no difference between groups. (TIFF) [file pone.0209704.s003.tiff]

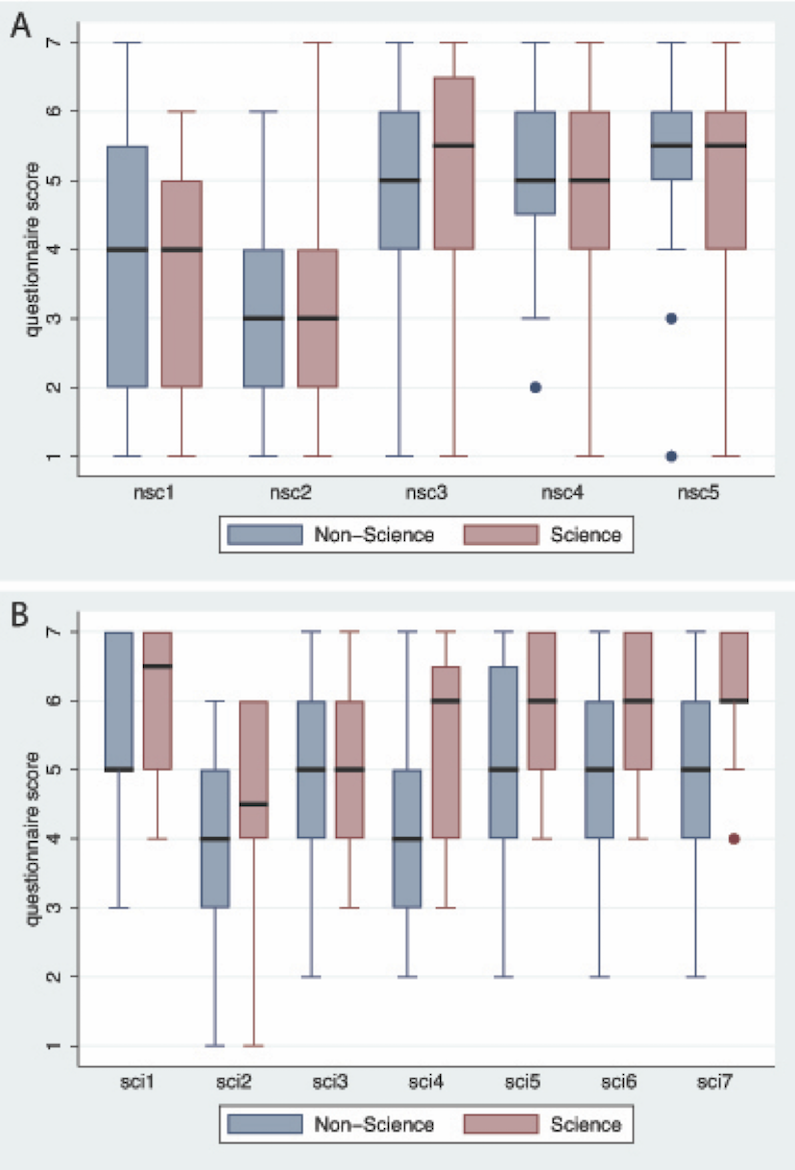

Supplement: S4 Fig — Box plots of responses to the manipulation of (a) the identification with students (i.e. non-science) questionnaire and (b) the identification with science questionnaire. The medians are the thick horizontal lines and the boxes show the interquartile ranges (IQR). The whiskers extend from max (min value, lower quartile—1.5*IQR) to min (max value, lower quartile + 1.5*IQR). Points outside of this are shown individually. (TIFF) [file pone.0209704.s004.tiff]

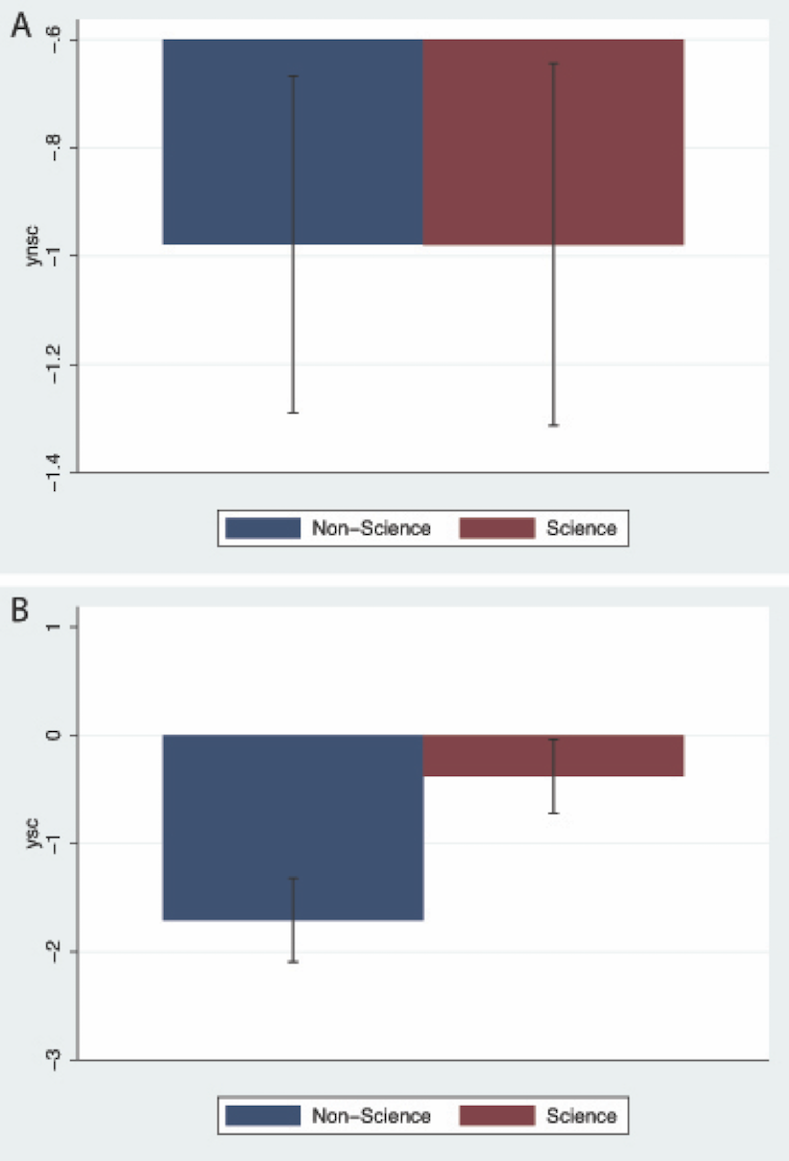

Supplement: S5 Fig — Bar charts showing the means and standard errors of the combined priming questionnaire score derived from the Polychoric PCA over the questionnaire scores. (TIFF) [file pone.0209704.s005.tiff]

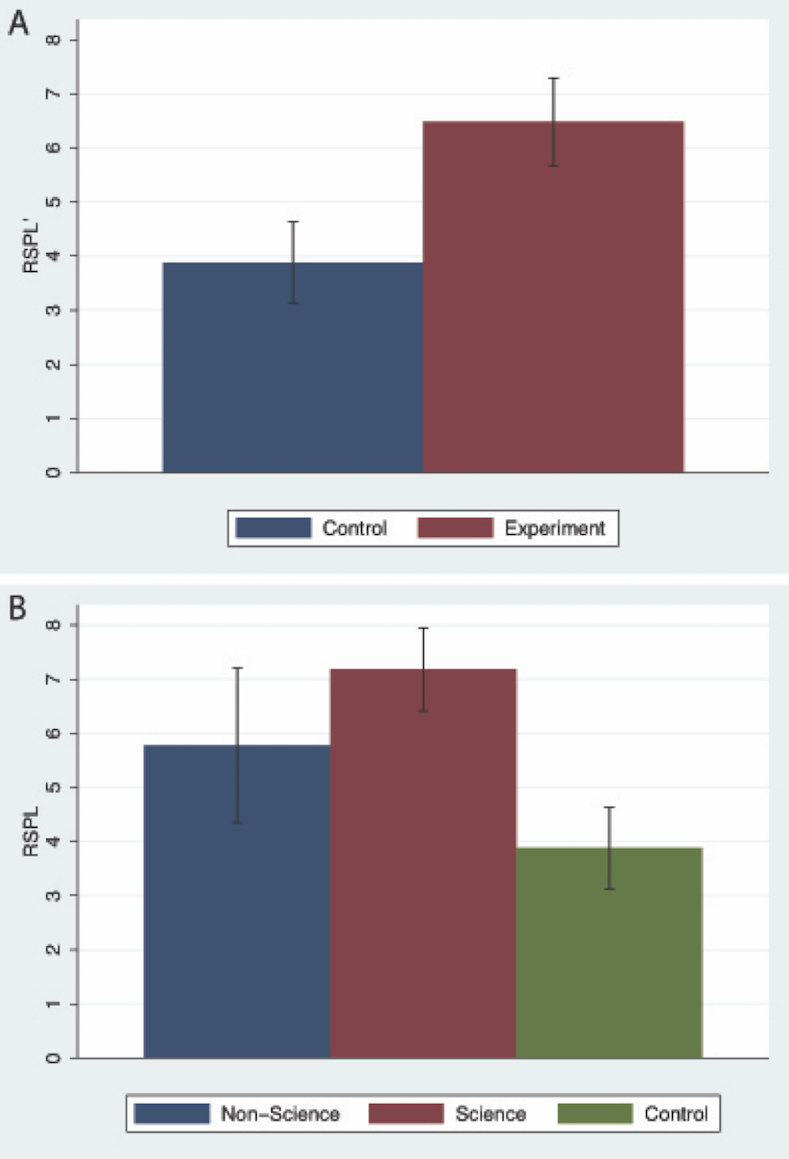

Supplement: S6 Fig — Bar chart showing mean ± SE of RSPL; (A) By the Control and Experimental (Science, Non-Science) groups (B) Distinguishing the Science and Non-Science groups. (TIFF) [file pone.0209704.s006.tiff]

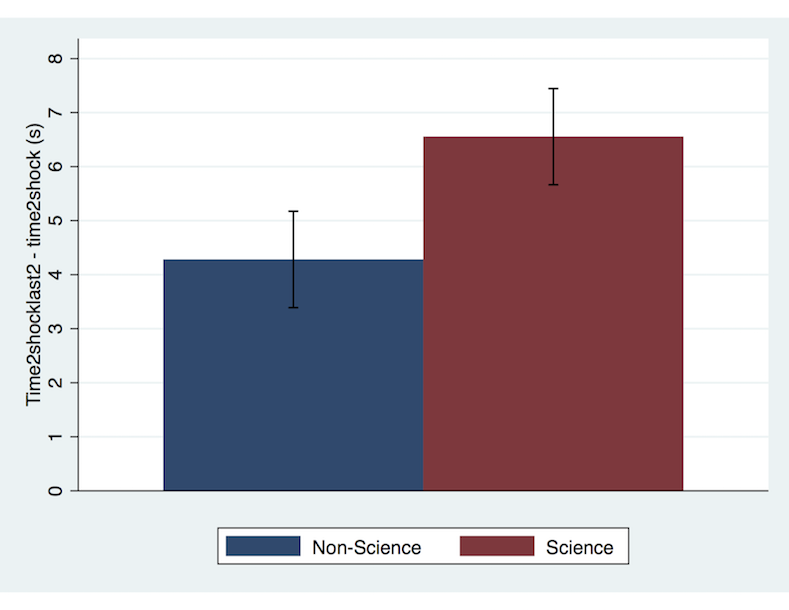

Supplement: S7 Fig — Difference between experimental conditions on the measure ‘timetoshock’. (TIFF) [file pone.0209704.s007.tiff]

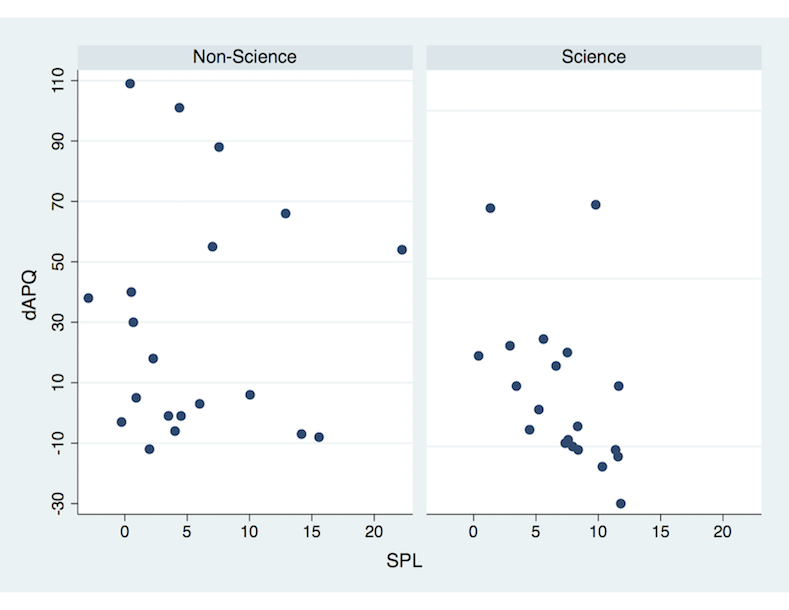

Supplement: S8 Fig — Scatterplot illustrating the relationship between helping and stress by experimental group. (TIFF) [file pone.0209704.s008.tiff]
