# Supplementary material for: Participant concerns for the Learner in a Virtual Reality replication of the Milgram obedience study
Source: PLoS One. 2018 Dec 31;13(12):e0209704. doi: 10.1371/journal.pone.0209704 (PMC6312327; doi:10.1371/journal.pone.0209704)
Supplement: S1 Text — Procedure used for randomising participants to conditions and the script followed by the avatar (i.e. the ‘Learner’). (PDF) [file pone.0209704.s009.pdf]

S1 Text

Random Assignment of Participants

In order to randomly assign the participants to either the Student or the Science conditions we first ran a randomizer (https://www.randomizer.org/) on an array of 40 values from 0 (student) to 1 (science), and made sure that the conditions were counter balanced. This way the conditions were pre-assigned even before the first participant arrived to take part in the experiment.

Script

The study followed a predefined set of options and interactions that were triggered by the experimenter who sat in a computer behind the participant (See S1Video). Some of the trials included the delivery of a shock by the participant. The response of the avatar to the shock was incremental. The complete sequence of the experiment used the following script:

Virtual Character (V): I'm ready to begin, are you also ready?
Participant (P): Yes
Experimenter (E): Please, read aloud.

1 Trial
P: Red, blue GREEN yellow black
V: Green
P: Correct

2
P: Cub, tiger fox BEAR panda
V: Bear
P: Correct

3
P: Tea, LEAF cup bag tray
V: Cup
P: Incorrect, the correct answer was Leaf
/// SHOCK

4
P: Pollen,grass flower TREE soil
V: Can you repeat that please?

5
P: Pollen, grass flower TREE soil
V: Flower
P: Incorrect, the correct answer was Tree
/// SHOCK

6
P: Road, avenue street LANE path
V: Avenue?
P: Incorrect, the correct answer was Lane
/// SHOCK

7
P: Wheel, car truck scooter BIKE
V: Bike
P: Correct

8
P: Ball, racket bat game BOY
V: Bat
P: Incorrect, the correct answer was Boy
/// SHOCK
V: Ahh

9
P: Ear, talk LISTEN plug telephone
V: Telephone
P: Incorrect, the answer was Listen
/// SHOCK
V: Aghh this really hurts

10
P:Bell, ring lamp bronze STRIKE
V: lamp
P: Incorrect, the answer was Strike
/// SHOCK
V: Ahh ahh

11
P: Soup, SPOON bowl can dish
V: spoon
P: Correct

12
P: Bean, GREEN coffee counter bag
V: Coffee
P: Incorrect, the answer was Green
/// SHOCK
V: aghh I don't want to continue

13
P: Inflate, pump balloon AIR ego
V: Speak Louder please,

14
P: Inflate, pump balloon AIR ego
V: Balloon
P: Incorrect, the answer was Air
/// SHOCK
V: ahh, I wanna go out

15
P: Banana, apple GRAPE orange lemon
V: Orange
P: Incorrect, the answer was Grape
/// SHOCK
V: ahhhh iiii, let me out I had enough

16
P: Giant, SIZE magic man squid
V: Forget it, I’m not answering
E: Remember that if he doesn’t answer the answer should be taken as incorrect
V: I don’t want to continue, don’t listen to her
// SHOCK
V: ahhh you have no right to keep me here, let me out

17
P: Brown,wood PEANUT carpet chocolate
V: Peanut
P: Correct

18
P: Sugar, cake sweet CUBE salt
V: Salt
P: Incorrect, the answer was Cube
/// SHOCK
V: ahhhh, I want to stop now

19
P: Jewellery, silver necklace ring GOLD
V: Necklace
P: Incorrect, the answer was Gold
/// SHOCK
V: ahhhh, let me out I’ve had enough

20
P: Water, fall MILK juice tea
V: juice?
P: Incorrect, the answer was Milk
/// SHOCK
V: ahhhhh, ah, I don’t want to continue

21
P: Seat, GROUND chair stool sofa
V: Sofa
P: Incorrect, the answer was Ground
/// SHOCK
V: ahhh, I didn’t agree to this

22
P: Road, avenue STREET lane path
V: avenue
P: Incorrect, the answer was Street
/// SHOCK
V: ahhh, please let me out, I want to stop

23
P: Tulip, daisy lilly carnation ROSE
V: Rose
P: Correct

24
P: Drink, CAN cup glass bottle
V: What?

25
P: Drink, CAN cup glass bottle
V: Can
P: Correct

26
P: Ear, Talk LISTEN plug telephone
V: telephone
P: Incorrect, the answer was Listen
/// SHOCK
V: ahhhhhhh, I’m not doing the experiment anymore

27
P: Tea, cup bag tray LEAF
V: cup
P: Incorrect, the answer was Leaf
/// SHOCK
V: ahhhhh...

28
P: Pollen, grass flower TREE soil
V: (no answer, fainted)
P: Incorrect, the answer was Tree
/// SHOCK
V: ahhhh

29
P: Bell, ring tower bronze STRIKE
V: (no answer, fainted)
P: Incorrect, the answer was Strike
/// SHOCK

30
P: Soup, bowl can SPOON dish
V: (no answer, fainted)
P: Incorrect, the answer was Spoon
/// SHOCK
