# Supplementary material for: Participant concerns for the Learner in a Virtual Reality replication of the Milgram obedience study
Source: PLoS One. 2018 Dec 31;13(12):e0209704. doi: 10.1371/journal.pone.0209704 (PMC6312327; doi:10.1371/journal.pone.0209704)
Supplement: S2 Text — Items and procedural information relating to the APQ stress measure. (PDF) [file pone.0209704.s010.pdf]

S2 Text

APQ Stress Measure

To measure self-perceived physiological arousal the APQ [22] was used. This was administered before the experiment(APQpre) and immediately after (APQpost). Examples include the following:

Awareness of many bodily sensations

very few | very many
|____________________________________|

Frequency of awareness of those sensations

never | throughout
|____________________________________|

Trembling or shaking

not at all | a great amount
|____________________________________|

Lack of concentration

full concentration | extreme lack of concentration
|____________________________________|

Dizziness

not at all | a great amount
|____________________________________|

There are 24 such questions, and each was scored on a 1-10 scale according to how far along the line the participant had marked their answer. Hence the maximum scores were 240 for each of APQpre and APQpost where the greater the score the greater the physiological arousal. Of interest are the differences in scores dAPQ = APQpost – APQpre.
