# Supplementary material for: Participant concerns for the Learner in a Virtual Reality replication of the Milgram obedience study
Source: PLoS One. 2018 Dec 31;13(12):e0209704. doi: 10.1371/journal.pone.0209704 (PMC6312327; doi:10.1371/journal.pone.0209704)
Supplement: S3 Text — Items measuring participants’ levels of familiarity with Milgram’s obedience studies. (PDF) [file pone.0209704.s011.pdf]

S3 Text

Familiarity with Milgram

Participants' prior familiarity with the Milgram paradigm was assessed by a series of questions resulting in a score from 0 (least) to 4 (most) familiarity. Also, recruiting was done in a way as to minimize the number of participants that would be familiar with Milgram's studies (e.g., psychology students were excluded).

The questionnaire contained the following questions:

1. Were you aware of similar studies? (yes/no)
2. Did you think of them while doing the experiment? (no/ yes but they did not affect me / yes and they affected me during the experiment)
3. What did they find in those experiments?
4. Do you remember who did them?
5. What was the message of those experiments?
6. Would you have continued to the end of all the trials if the situation had been real (in other words, if the character had been real) would you have been likely to continue through all the voltages to the end of all the trials? (from 1 certainly not / to 7 certainly yes).
7. I estimate that the proportion of people who would carry on to the highest voltages would be x/100. If the situation had been real (in other words, if the character had been real), how many people out of 100 do you believe would have carried on to the highest voltages in spite of the protests and discomfort of the person who was being shocked.

The responses were compiled resulting in a score from 0 (least) to 4 (most) familiarity, that consisted of the sum of Q1+Q2+Q3+Q4. Maximal familiarity would also be achieved by participants who remembered who did the experiments and what was found. Q5 was a consistency check of Q4.
