# Supplementary material for: Participant concerns for the Learner in a Virtual Reality replication of the Milgram obedience study
Source: PLoS One. 2018 Dec 31;13(12):e0209704. doi: 10.1371/journal.pone.0209704 (PMC6312327; doi:10.1371/journal.pone.0209704)
Supplement: S4 Text — Explanations, items, and analysis of the questionnaire used to assess the efficacy of the priming. (Table 1) Items used to assess priming efficacy. (Table 2) Correlations for priming scores. (PDF) [file pone.0209704.s012.pdf]

S4 Text

The Priming Questionnaire

To assess the efficacy of the priming a questionnaire was administered after the exposure that consisted of a set of 7 questions that assessed the degree of identification with science, and a further 5 questions to that assessed the degree of identification with students.

Table 1. Items Used to Assess Priming Efficacy.

**Variable name**	**Question**
sci1	I feel good about furthering psychological science
sci2	I identify with the researchers who designed this study
sci3	I identify with the goals of psychological science
sci4	I feel positive about the scientists behind this research
sci5	I feel strong ties with people who are pro-science.
sci6	I think of myself as part of the pro-science community
sci7	I identify with supporters of science.
nsc1	I identify with the learner in this study
nsc2	I fell positive about the learner in this study
nsc3	I feel strong ties with other students living in London.
nsc4	I think of myself as part of the student community in London.
nsc5	I identify with other students living in London

Each item was scored on a 1-7 scale, with 1 indicating no agreement and 7 indicating complete agreement.

In order to obtain one overall score for each of these we used polychoric principal components analysis [32]. This is a principal components analysis for ordinal scales, assuming that the ordinal scales are samples from an underlying continuous (latent) variable. In the case of Science

the first principle component (ysci) explained 57% of the variation in the 7 ordinal scores. Similarly the first principal component for the Students scores (ynsc) explained 58% of the variation of the 5 scores.

Table 2. Correlations for the Priming Scores

Variable	Spearman Correlation with ystu (n = 40)	Spearman correlation with ysc (n = 40)
nsc1	0.34	
nsc2	0.18	
nsc3	0.89	
nsc4	0.94	
nsc5	0.90	
sci1		0.62
sci2		0.44
sci3		0.50
sci4		0.55
sci5		0.92
sci6		0.93
sci7		0.94
