# Supplementary material for: Participant concerns for the Learner in a Virtual Reality replication of the Milgram obedience study
Source: PLoS One. 2018 Dec 31;13(12):e0209704. doi: 10.1371/journal.pone.0209704 (PMC6312327; doi:10.1371/journal.pone.0209704)
Supplement: S5 Text — Background information, items, and effect sizes of presences measures. (Table 1) The full set of presence questions and effect sizes comparing the two groups. (PDF) [file pone.0209704.s013.pdf]

S5 Text

Presence: Background on Measure

A questionnaire on presence was administered after the APQ. This measure has two components: the illusion of being in the virtual place (Place Illusion; PI), and the illusion that the events are taken as real (Plausibility; Psi) [33]. PI is thought to be largely influenced by the degree to which normal sensorimotor contingencies are afforded in the virtual environment. Plausibility, however, relies on prior experience, expectations, ecological validity, responsiveness of the actors in the scenario, etc. PI was assessed using 5 items, and Psi by 11 items. The full set of questions is shown in Table 1 below. In addition, this table shows effect sizes and the proportion of values of Non-Science > values of Science.

Table 1. The full set of presence questions and effect sizes comparing the two groups

Variable	Question	**Effect Size = Proportion of values of Non-Science > values of Science (= total sample size * (Mann-Whitney U statistic) PI**	**Effect Size = Proportion of values of Non-Science > values of Science (= total sample size * (Mann-Whitney U statistic) PSI**
there	I had a sense of being there in the training room	0.52	
real	There were times during the experience when training room was the reality for me	0.44	
visited	The training room seemed to be more like somewhere I visited	0.40	
lab	I had a stronger sense of being in a lab	0.63	
Invrlab	During the experience I was thinking that I was really in the VR laboratory	0.32	
realsituation	I responded as if the situation were real		0.49
realplace	I responded as if it were a real place		0.56
realemotion	My emotional response in the training room was the same as if it had been real		0.51
realthoughts	My thoughts with in the training room were the same as if it had been real		0.38
realbehave	In spite of my knowledge that the situation wasn't real I found myself behaving as if it were real		0.45
realphysical	My physical responses within the training room were the same as if it had been real		0.51
hereal	I behaved as if he as if he were real		0.46
herealemotion	My emotional response to him was as if he were real		0.35
herealthoughts	My thoughts in relation to him were as if he were real		0.47
hrealphysical	My physical responses to him were as if he were real		0.48
herealbehave	In spite of my knowledge that he wasn't real I found myself behaving as if he were real		0.35

There may be some tendency for those in the Science group to have higher scores than those in the Non-Science group. However, 11/16 proportions are in the range 40-60%, and 9/16 between 45-55%.
