# Supplementary material for: Participant concerns for the Learner in a Virtual Reality replication of the Milgram obedience study
Source: PLoS One. 2018 Dec 31;13(12):e0209704. doi: 10.1371/journal.pone.0209704 (PMC6312327; doi:10.1371/journal.pone.0209704)
Supplement: S6 Text — Items and effect sizes for items measuring participants experiences with gaming, computers, programming, and VR. (Table 1) Questions and their effect sizes. (PDF) [file pone.0209704.s014.pdf]

S6 Text

Familiarity with technology

Here we compare the effect sizes of the difference between groups. Both the effect sizes (which correspond to chance values) and the box plots demonstrate that there were no prior differences between groups on their level of familiarity with technology.

Table 1. Questions and their effect sizes

**Variable**	**Question**	**Effect Size = Proportion of values of Non-Science > values of Science (= total sample size * (Mann-Whitney U statistic)**
Gamer	Which category of computer gaming would you say applies most to you?<br>**0 -** Non-Gamer: I rarely or never play computer games.<br>**1 -** Casual Gamer: I play games for enjoyment and relaxation rather than games with steep learning curves or requiring high levels of commitment or involvement.<br>**2 -** Core Gamer: I typically play games with a steeper learning curve or games that require some level of deeper involvement or complex tactical challenges.<br>**3-** Hard-Core Gamer: I typically play high-action, extremely competitive games that require a greater degree of involvement or dexterity in order to progress.	0.58
Computer	Please state your level of computer literacy on a scale of 1 (novice) to 7 (expert)	0.59
Programming	Please rate your level of experience with computer programming on a scale of 1 (novice) to 7 (expert)	0.50
VR	Have you ever experienced 'virtual reality' before? on a scale of 1 (no experience) to 5 (extensive experience)	0.50
