# Supplementary material for: Participant concerns for the Learner in a Virtual Reality replication of the Milgram obedience study
Source: PLoS One. 2018 Dec 31;13(12):e0209704. doi: 10.1371/journal.pone.0209704 (PMC6312327; doi:10.1371/journal.pone.0209704)
Supplement: S1 Statsmodel — (PDF) [file pone.0209704.s017.pdf]

```
data {
    int<lower=0> n;
    int<lower=0> m;
    vector[m] student;
    vector[m] science;
    vector[m] cwordspl;
    vector[n] cond;
    vector[n] dapq;
    vector[n] wordspl;
    vector[n] condword;
    vector[n] ysci;
    vector[n] ystu;
    vector[n] time2shock;
    vector[n] time2shocklast2;
}

parameters {
    vector[2] aword;
    real<lower=0> sigma_word;

    vector[4] aapq;
    real<lower=0> sigma_apq;

    vector[3] atime;
    real<lower=0> sigma_time;

    vector[2] asci;
    real<lower=0> sigma_sci;

    vector[2] astu;
    real<lower=0> sigma_stu;

    vector[3] acword;
    real<lower=0> sigma_cword;
}

model {
    real s;
    real s2;
    s = 10;
    s2 = 5;

    for(i in 1:2) {
        aword[i] ~ normal(0,s);
        acword[i] ~ normal(0,s);
        aapq[i] ~ normal(0,s);
        atime[i] ~ normal(0,s);
        asci[i] ~ normal(0,s);
        astu[i] ~ normal(0,s);
    }
```

```
    for(i in 3:4) {
        aapq[i] ~ normal(0,s);
    }
    atime[3] ~ normal(0,s);
    acword[3] ~ normal(0,s);

    sigma_word ~ cauchy(0,s2);
    sigma_cword ~ cauchy(0,s2);
    sigma_apq ~ cauchy(0,s2);
    sigma_time ~ cauchy(0,s2);
    sigma_sci ~ cauchy(0,s2);
    sigma_stu ~ cauchy(0,s2);

    wordspl ~ normal(aword[1] + aword[2]*cond, sigma_word);
    cwordspl ~ normal(acword[1] + acword[2]*student +
acword[3]*science,sigma_cword);
    dapq ~ normal(aapq[1] + aapq[2]*cond + aapq[3]*wordspl +
aapq[4]*condword,sigma_apq);
    time2shocklast2 ~ normal(atime[1] + atime[2]*time2shock +
atime[3]*cond,sigma_time);
    ysci ~ normal(asci[1] + asci[2]*cond,sigma_sci);
    ystu ~ normal(astu[1] + astu[2]*cond,sigma_stu);
}

generated quantities {
    vector[n] wordspl_new;
    vector[n] dapq_new;
    vector[n] time2shocklast2_new;
    vector[n] ysci_new;
    vector[n] ystu_new;
    vector[m] cwordspl_new;

    for(i in 1:n) {
        wordspl_new[i] = normal_rng(aword[1] + aword[2]*cond[i],
sigma_word);
        dapq_new[i] = normal_rng(aapq[1] + aapq[2]*cond[i] +
aapq[3]*wordspl[i] + aapq[4]*condword[i],sigma_apq);
        time2shocklast2_new[i] = normal_rng(atime[1] +
atime[2]*time2shock[i] + atime[3]*cond[i],sigma_time);
        ysci_new[i] =  normal_rng(asci[1] +
asci[2]*cond[i],sigma_sci);
        ystu_new[i] =  normal_rng(astu[1] +
astu[2]*cond[i],sigma_stu);
    }
    for(i in 1:m)
        cwordspl_new[i] = normal_rng(acword[1] + acword[2]*student[i]
+ acword[3]*science[i],sigma_cword);
}
¥
```
